# Analyzing the mechanism of strategic orientation towards digitization and organizational performance settings enduring employee resistance to innovation and performance capabilities

**DOI:** 10.3389/fpsyg.2022.1006310

**Published:** 2022-09-29

**Authors:** Yurong Wang

**Affiliations:** ^1^School of Business, Xi’an University of Finance and Economics, Xi’an, China; ^2^The Research Center of Modern Enterprise Management, Xi’an University of Finance and Economics, Xi’an, China

**Keywords:** resistance to innovation, strategic orientation, innovative organizational performance, digital knowledge capability, innovation capability

## Abstract

Resistance to innovation is a behavioral barrier to implementing innovation in any organization. It is associated with employees’ demotivation to adopt new technologies. Strategic orientation toward digitalization is a new dimension in shaping innovative organizational performance. It is also evident from past studies that certain employees’ capabilities are associated with organizations’ strategic orientation when undergoing digitalization. This study examines the relationship between these factors and achieving innovative organizational performance. First, it looks at how strategic orientation toward digitalization relates to digital capabilities, which include digital knowledge and innovation. This study also examines how capabilities affect strategic orientation toward digitalization and innovative organizational performance. Moreover, the negatively regulating role of resistance to innovation as a moderator is also tested between capabilities and innovative organizational performance in this research. The authors discovered a connection between strategic orientation towards digitalization and innovative organizational performance in their research. A Likert scale with five points was used to quantify the responses, and the points ranged from 1 to 5 on the scale, with one being strongly disagreed and five strongly agreed. The findings of the study also show that digital knowledge capability and innovation capability have a substantial impact on innovative organizational performance. The research also discovered that employees’ resistance to innovation exerts a sizeable moderating influence on the connection between digital knowledge competence and innovation capability within the innovative organizational performance. The study’s results show that businesses must have a strategic focus on digitalization if they want to improve their ability to come up with new ideas and their digital knowledge skills, which are both critical for the growth of the entrepreneurial system. The model that has been proposed is available to entrepreneurs so that they can apply it in their businesses to advance the entrepreneurial system appropriately. The authors present a theoretical model for entrepreneurial systems based on the strategic orientation towards the digitalization approach. This model is something that entrepreneurs could utilize to improve the performance of their organizations as a whole. In addition, the employee’s resistance to innovation is used as a moderator in the model, which is another innovative method. The research contributes new and essential information to the existing literature on innovative organizational performance.

## Introduction

Researchers in marketing, management, and innovation studies have focused much attention on how businesses are oriented strategically. Even though numerous typologies and frameworks for strategic orientations have been created across different literature streams, the notion typically refers to “principles that control and affect the operations of an organization and generate the behaviors meant to ensure its existence and innovative performance” (Masadeh et al., 2018, p. 3117). The concept’s core idea is that businesses should be focused on identifying, gathering, and analyzing information to generate new knowledge within their organizations (Senanu and Anning-Dorson, 2022). Therefore, strategic orientation may be crucial to an organization’s innovation process. According to evolutionary economics, new knowledge presents chances to develop new ideas along known trajectories, combine knowledge in novel ways, and produce novel paths for innovation (Arant et al., 2019). The direct impact of strategic orientation on organizational performance is examined in a large portion of the empirical research that is now available on strategic orientations (Adams et al., 2019). The literature on marketing’s relationship between performance and customer/market orientation was the basis for past research. However, it was expanded in other literature streams to include more orientation types (for example, technology orientation and entrepreneurial orientation) and a more narrow focus on innovative organizational performance (Adams et al., 2019).

An organization’s strategic orientation is the direction it takes to ensure that its actions are appropriate to attain high performance (Hutahayan, 2021). It is a collection of principles that make up a company’s strategic direction (Brooksbank et al., 2018). Strategic orientation is therefore essential for improved organizational performance. According to Hutahayan (2021), strategic orientation enhances business innovation and marketing success. It serves as a set of guiding principles that the organization may rely on throughout tumultuous times. It outlines the objectives of the business. As a result, each organization has a particular strategic approach. For example, a firm striving to have a strong client orientation would have a different strategic orientation than an organization concentrating on innovation.

If the business is concerned with cost savings, its strategic focus would be on bolstering its value chain (Mwenda, 2020). The management of capital for the growth of innovative products and services would emphasize the company’s strategic orientation with a goal of competitive advantage (Sahi et al., 2020). Similarly, firms with a strategic resource orientation create unique resources and utilize natural resources (Colclough et al., 2019). The strategic focus on digitization is on a business’s transition to the digital age. Due to the advancement, businesses worldwide are rapidly adopting digitalization, making this form of strategy orientation special. Customers choose to make purchases from businesses that use digital technology and satisfy their requirements and wants both now and in the future. The strategic focus on digitization helps customers to make quick, simple, and satisfying purchases of goods and services (Brooksbank et al., 2018). Companies that transition to meet industry expectations integrate operations, human resources, innovation, and consumer solutions, claims strategy. Due to the broad customer demand in this digital era, digitalization of strategic orientation also forces enterprises to adopt various products and services. Due to the present pandemic, businesses must adjust their strategic orientation toward digitalization to compete in the market (Colclough et al., 2019). External factors and product qualities may indicate whether one strategic orientation is better than another.

The empirical research, in contrast, has paid comparatively little attention to the variables that might mediate the association between strategic orientation towards digitalization and innovative organizational performance (Genc et al., 2019). Preliminary research suggests that organizational factors mediate this association (Zhang and Duan, 2010). Little is known about the impact of what effective organizations are doing and how they use their resources to address the knowledge assets in mediating their performance. As a result, there is a knowledge gap in the research community regarding how knowledge and innovation capability may mediate the relationship between strategic orientation towards digitization and innovative organizational performance (Rajapathirana and Hui, 2018). Organizational innovation must overcome several obstacles, including raising money, having enough infrastructure and equipment, training current employees, finding the right people to hire and keep on staff, planning for employee and supplier servicing, and expanding and migrating to new communication channels. The uniqueness of innovative organizational performance is that. In contrast, technology innovation is readily accepted in daily life on a social level; its application at work causes employee dissatisfaction and is reluctantly accepted (Župerkienė et al., 2019). The major hindrance to innovative processes of the organization lies in the resistance to innovation by employees and other stakeholders.

Researchers have noted that one of the most frequent reasons for failing to implement innovation is employees’ resistance to innovation (Alcover et al., 2022; Bohata et al., 2022). Employee resistance to innovation caused almost 70% of organizations to fail to implement innovation programs (Shahbaz et al., 2019). Instead of concentrating solely on technical aspects while implementing innovation within the organization, managers must also consider the human aspects of innovation (Bucea-Manea-Țoniş et al., 2021). Researchers agree that a significant or crucial stage of the organizational transformation process involves lowering resistance to innovation (Ghobadian et al., 2020). Moreover, these researchers identified that employees’ resistance to change had not been studied before as a moderator between strategic orientation towards digitalization and innovative organizational performance.

To fill this research gap and keep in view its negative role in the innovation process, the following research utilized employees’ resistance to innovation as a moderator hindering the innovative organizational performance. As discussed earlier, specific individual capabilities like employees’ digital knowledge and innovation capabilities may mediate these relationships (Chaudhry et al., 2019; Chaudhuri et al., 2022). In the past, no research has evaluated mediating roles of digital knowledge and innovation capabilities between strategic orientation towards digitalization and innovative organizational performance. Therefore, current research fills this gap by evaluating mediating roles of these individual capabilities of employees. This notion is based on the Resource base view (RBV) of the firm’s theory having an extension to it as dynamic capabilities. This theory underpins the justification of capabilities resources in innovative organizational performance. Khin and Ho (2020) indicated that the organizations lacking employees’ dynamic capabilities show limited transformation towards digitalization and innovative performance. Therefore, it is necessary to evaluate the dynamic capabilities of employees in this research, which are digital knowledge and innovation capabilities. The current research addresses the following questions: Do the organizations need strategic orientation towards digitalization for attaining innovative performance? What role resistance behaviour of employees towards innovation would play in this performance?

Moreover, what are the underlying mediating mechanisms in this sort of innovative performance of organizations? To address these questions, the following research evaluates the association between strategic orientation towards digitalization and innovative organizational performance. This research further evaluates the mediating roles of digital knowledge and innovative capabilities of employees between strategic orientation towards digitalization and innovative organizational performance. The research also finds the regulating role of employees’ resistance to innovation in organizational innovation. The research contributes significantly to the regional firms and business organizations developing strategic orientation toward digitalization. The subsequent sections of this article emphasize the literature review, methodology, results, discussions, and conclusions of the research.

## Literature review

### Theoretical support and hypotheses development

Recent investigations from the late 1990s support the theoretical literature on the topic of strategic orientation towards digitalization. For example, Ahmad et al. (2013) research defines strategic orientation as the organizational choice, trust, fit, and design. Three methods can be used to test it: comparison (identify traits), classification (apply conceptual bias), and narrative (use qualitative research). The ideas around digitization focus on creating long-term development plans to maintain market competitiveness. Two perspectives—the resource-based view and the core competency view—are used to describe these theories. The resource-based perspective stresses the value of digital transformation and using extra resources (Kim and Kim, 2022).

According to this perspective, the degree of employee participation determines the extent of digitalization because it integrates the operational and management resources of the company, boosting its market value (Zeshan et al., 2021). Additionally, this perspective asserts that companies should spend their resources following how the external environment is changing so that they may remain competitive (Mikalef et al., 2021). On the other hand, the core-competence theory holds that an organization’s ability to compete in the market depends on how effectively and efficiently it allocates its resources and engages in R&D activities. Digitalization allows data to flow automatically, reducing interruptions and boosting resource allocation efficiency (Yu and Moon, 2021). The strategic focus on digitization transforms company models, restructures its systems, and fosters innovation (Satalkina and Steiner, 2020). This research is backed up by a resource-based view of the firm’s theory. It suggests that organizations must have specific resources like orientation and dynamic capabilities of employees to achieve innovative organizational performance. The dynamic capabilities of employees include but are not limited to digital knowledge capability and innovation capability. So, the dynamic capability theory under RBV provides conceptual support to the mediators of this research.

Moreover, this research gets support from RBV in the context of employees’ resistance to innovation. Resistance to innovation is considered a barrier to achieving innovative performance. Considering it as a strategic resource, emphasis should also be placed on eliminating this behavior among employees. Hence, it would add to the human resource of the organization.

### Strategic orientation towards digitalization and capabilities

Strategic management, entrepreneurship, and marketing all regularly use the idea of strategic orientation (Falahat et al., 2018). Strategic orientation of firms is also considered an aspect of business management for the flourishing of businesses. The business’s strategic decisions and compatibility with the environment are seen through the lens of strategic orientation (He et al., 2020). An organization’s strategic orientation assesses how it acquires, uses, and distributes resources to develop dynamic capabilities (Puspaningrum, 2020). The Resource-Based View (RBV) theory also contends that better company performance, competitive edge, and strategic success depend on an organization’s resources. To achieve a competitive edge and extraordinary profits, a company needs to recognize and utilize resources that are precious, unique, difficult to duplicate, and non-replaceable (Barney, 1991).

Researchers like Fonseca (2021) and others have viewed RBV theory as the foundational management theory for business excellence. Businesses that are focused on digitalization prioritize technologies, goods, or processes. In reality, this approach is frequently contrasted with a customer orientation to show significant variations in viewpoints on the primary source of customer value (Adams et al., 2019). Customer value is produced by new solutions based on technological breakthroughs rather than customer inputs for businesses with a technology orientation (Wang et al., 2022). Typically, this perspective entails a significant commitment to R&D initiatives that seek to explore and learn about emerging technology. Digitalization-oriented businesses attempt to learn new technologies and apply them to create innovative goods and procedures (Du et al., 2016). Empirical studies often strongly correlate a digital technology focus and business performance. These studies also imply that if market and technology instability increases, an orientation towards digitalization may have a more favorable effect on performance (Truant et al., 2021). Surprisingly, however, there has not been as much empirical research to look at the direct link between digitalization and the success of innovations. However, Du et al. (2016) studied new product creation in industrial markets and provided evidence for a good link.

According to Cucculelli et al. (2016), firms’ production and technological competence propel innovative items to commercial success in these marketplaces. Studies on innovation also show that exposure to sources of technical opportunity and technological expertise are essential components of successful product developments (Segarra-Ciprés and Bou-Llusar, 2018; Bello-Pintado and Bianchi, 2020). The extant study emphasizes how skills and information gained from R&D may influence the design of innovations and their successful commercialization (Guo et al., 2016). All this supporting literature on the role of strategic orientation towards digitalization helped us formulate a basis for innovative organizational performance.

However, many businesses engaged in digital transformation are still unsure how to create their digital organizations, grow the skills and tools necessary to manage digital information, create and maintain online services, and automate. Most businesses start without a solid understanding that many essential resources required to assist digitalization will not be accessible on the company’s premises. At the same time, it is imperative that many industries transition to a digital economy; doing so internally can take years. However, in the long run, this strategy can assist businesses in overcoming the obstacles of innovation and improving their ability to compete online. At the same time, acquiring digital talents from the outside is probably tricky (Rupeika-Apoga et al., 2022). One of the requirements of digitalization is the creation of the capabilities required in various fields. Still, the diversity of capabilities relies on the particular industry and the particular business demands (Saputra et al., 2022). Digital capabilities influence digital innovation favorably, which leads to digitalization (Bullini Orlandi, 2016). These capabilities are of many types, including digital knowledge and innovation. These capabilities are dynamic and contribute significantly to innovative organizational performance. The direct relationships of strategic orientation towards digitalization with digital knowledge capability and innovation capabilities of employees are tested in this study as per the following hypotheses.

*H1*: Strategic orientation towards digitalization positively correlates with digital knowledge capability.

*H2*: Strategic orientation towards digitalization has a positive association with innovation capability.

### The mediating role of capabilities of employees

Digital literacy goes well beyond merely learning how to operate a computer. The ability to comprehend and utilize technology in a society that is becoming more connected is known as having digital knowledge (Reddy et al., 2020). Employees must understand how their online interactions can have a lasting effect on both their personal and professional lives. Technological knowledge acquired in one’s primary field of study is required, but gaining soft skills, such as digital awareness, is also crucial. When forced to operate in an environment that is tough to use digitally, employees with inadequate digital awareness skills only know little. Such an individual will have difficulty with Gmail, some cloud technologies, social networking applications, and other programs that require a thorough understanding of their user interface and the underlying setups for security considerations (Gfrerer et al., 2021).

Understanding one’s online identity entails taking steps to preserve one’s privacy online, improve computer security, use social media and networking responsibly, and safeguard one’s digital reputation and footprint (Murthy and Gopalkrishnan, 2022). Innovation capability is a term used to describe a company’s ability to develop new and improved products and services. These innovative capabilities result from three factors: clients, marketing, and technology (Vu, 2020). The ability to quickly and differently mobilize innovative solutions and services than rivals is known as the client dimension; the implementation of novel marketing strategies is known as the marketing aspect; and the company’s goal of utilizing the most recent software, structures, and software products to improve primarily its overall operations is known as the technology dimension (Mikalef and Krogstie, 2020). As a result, the connection between strategic orientation towards digitalization efforts and innovation capability is unmistakable. Corporate organizations will be better able to produce new and enhanced products, services, and processes if they seek to identify technology breakthroughs and recognize the digitalization process (Saunila, 2020). As the organizations think of methods to increase value from digitalization, the consumer experience will be improved by adding more interactive tools to the current product offering, as shown by the growth of financial services (Vu, 2020). It is evident from the literature that the strategic orientation of firms towards digitalization also emphasizes developing specific stakeholders’ digital and innovation capabilities. It is also assumed that such capabilities help in achieving innovative organizational performance. Therefore, the following hypotheses were developed to test the mediating roles of these capabilities.

*H3*: Digital knowledge capability mediates the relationship between strategic orientation towards digitalization and innovative organizational performance.

*H4*: Innovative capability mediates the relationship between strategic orientation towards digitalization and innovative organizational performance.

### Moderating the role of employees’ resistance to innovation

Ridley (2020) asserts that a condition of innovation is one in which there are distinctions between new and conventional ways of thinking. Employee resistance to innovation (ERI) refers to a person’s behavior that shields them from the effects of either real or imagined change (Mani and Chouk, 2018). ERI is described by Chen et al. (2022) as the maintenance of the existing quo through individuals instigating opposition to the novel system. Due to its view as a potential threat to the stability of ingrained routines, every novel system frequently causes anxiety and carries ERI (Chua, 2018). Drosos et al. (2021) suggested that the organization should encourage staff to learn new skills, tasks, and programs to prevent ERI in workplaces when installing new systems or methods of working. ERI is a crucial component of personality and significantly impacts technology adoption (Mani and Chouk, 2018).

According to earlier research, ERI is a kind of demotivation that has been found to have a detrimental impact on people’s willingness to use information technology (Wang and Pan, 2022). According to Alomari et al. (2014), ERI was one of the factors that led to the non-adoption and failure of new information systems. By focusing on ERI, Lallmahomed et al. (2017) evaluated the adoption behavior of an e-government system and found a substantial inverse link between ERI and adoption. Many other researchers demonstrated the importance of ERI, and negative connections with acceptance of information and communication technology systems were discovered (e.g., Abbas et al., 2017). ERI was explored as a moderator between BIs and the adoption of green supply chain management (Shahbaz et al., 2019). It was found that higher ERI among employees would result in the non-implementation of GSCM.

Similarly, Beal III et al. (2013) looked at the moderating effect of ERI between organizational citizenship conduct and psychological capital. ERI is likely to strike a balance between deliberate actions and actual employee use of the BDA system in healthcare organizations. According to earlier research, ERI either had little or no direct impact on how technology was used (Beal III et al., 2013). As a result, rather than concentrating on ERI’s direct influence on actual use, this study additionally emphasizes the moderating role of ERI. These studies indicated that resistance to innovation is a moderator that negatively influences the innovation process. Therefore, the current study explores its negatively moderating role between digital capabilities and innovative organizational performance.

*H5*: Employee resistance to innovation moderates the relationship between digital knowledge capability and innovative organizational performance.

*H6*: Employee resistance to innovation moderates the relationship between innovative capability and organizational performance.

**Figure 1:**
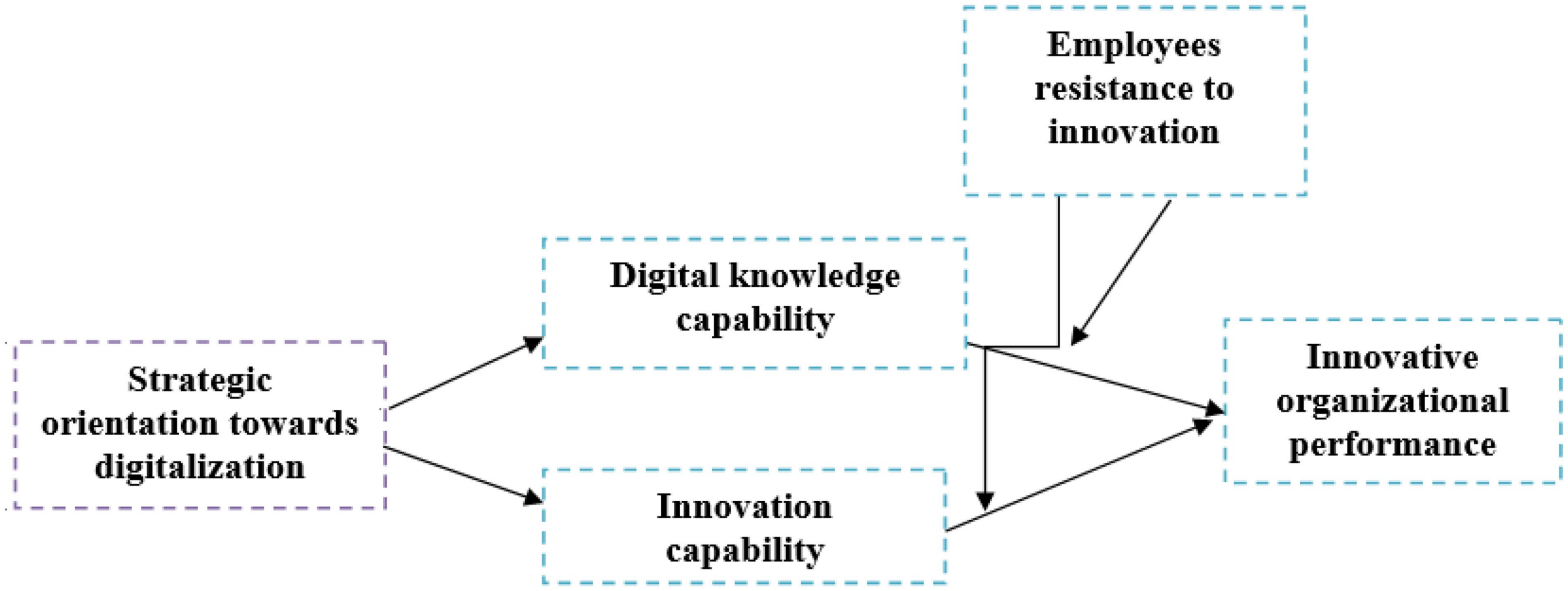
Conceptual framework.

The questions were designed to reflect how well the respondents understood the concepts of digitalization knowledge (Chaudhuri et al., 2022). The present study’s conceptual framework is given in Figure 1.

## Research methods

We relied on the survey methodology in this study to validate the model. We produced a set of questions to test the content validity of the constructs by drawing on the knowledge gathered from the previously published literature, theories, and scales already in use. Industry professionals who are highly knowledgeable in this field provided their input, which helped significantly expand the investigation’s scope. It was emphasized that questions should not be construed as leading or unclear to maximize the number of responses (Chaudhuri et al., 2022). Five sections with several questions were prepared and presented as statements. Each respondent was instructed to place one tick mark in one of the possibilities listed on the response sheet, which contained five options for each question. A Likert scale with five points was used to quantify the responses, and the points ranged from 1 to 5 on the scale, with one being strongly disagreed and five being strongly agreed (SA; Chaudhuri et al., 2022).

### Data collection

To reach the possible respondents, we contacted the top officials of the companies by phone and email. We asked them to allow managers of varying ranks to participate in the survey. We were successful in reaching our target audience. We gave these high-ranking authorities our word that the research would be kept entirely confidential and that it would be used only for academic purposes. After some initial reluctance, the top officials of 20 startup businesses eventually permitted managers of varying positions to participate in this poll. They subsequently forwarded us a list of managers along with their respective contact information.

It was not feasible to get information on the whole of the professional community in China, a method known as stratified random sampling was employed in order to choose respondents from within that particular category. If the total number of people in the population is unknown, one may still establish the appropriate sample size for survey research by using the following methodology, as suggested by (Rosenbaum and Spears, 2009; Molwus et al., 2017; Sahoo, 2019): The formula for determining the sample size is as follows: take the minimum required sample size and multiply it by 100. Then, divide that number by the projected average percentage of responders. To complete this survey’s objectives and achieve the conditions for the analysis, a minimum of 50 responses was necessary. Based on an expected 25 % response rate, determined from the standard response rate, the sample size for the present research was 500 participants. The survey link was sent to 500 professionals based in China. Following three reminders, each of which was given a gap of 15 days between them, there were 340 replies obtained. This accounts for 68% of the total respondents who received emails. 40 of the 340 replies provided were deemed inadequate. It was determined that 300 responses, equivalent to 60 percent of the total number of contacted respondents, were suitable for further analysis and acceptance.

After that, an analysis was performed using the replies from 300 respondents compared to 5 other sections. Three hundred respondents are within the acceptable margin of error (Arias-Pérez et al., 2020; Ardito et al., 2021; Chaudhuri et al., 2022). The process we opted for is straightforward and produces more fruitful outcomes in exploratory research. We employ the partial least square (PLS) structural equation modelling (SEM) method to analyze the findings of the responses to the quantitative questions. The PLS-SEM technique can perform a straightforward analysis of a complicated model and an analysis of data that is not regularly distributed (Chaudhuri et al., 2022). This cannot be accomplished using a covariance-based structural equation modeling approach. In addition, this method does not impose any restrictions on the samples (Robbins and Barnwell, 2006; Niemand et al., 2017, 2021; SANNES and Andersen, 2017; Satalkina and Steiner, 2020).

## Results

Smart PLS software can really come in handy when managing projects. One of the biggest benefits is that it saves a lot of time. Another benefit is that projects are tracked from start to finish so it’s easy to see what needs completion or what tasks need doing in order for your project plan to run smoothly. Another advantage of using smart PLS software is that it reduces or eliminates errors because everything is entered into an easy-to-read format. Users will only have to click on Submit once when entering information; this eliminates any chance of duplicating data or incorrect entries. Smart pls also does not require much training since its interface looks similar to popular operating systems like Windows or Mac OS X and making small changes can be done quickly by clicking on Options from the top menu bar. With regular use, many people find it easier to remember where certain functions are located within this system.

Though there are a few disadvantages to smart pls software, they are offset by the benefits. One downside is that it takes more time than traditional software because information needs to be entered into each tab instead of just the tabs necessary for one part of the project. Another negative aspect is that updates are not as frequent, which can cause problems if new changes need to be made that do not come with a newer version. There may also be mistakes during input if users do not double-check every keystroke before submitting their work. A final downside is that while parts libraries can be used with this type of software, some models cannot be imported in order to avoid conflicts between different brands. These limitations mean that an entire product cannot be modeled, but the advantages still outweigh these shortcomings.

### Correlation matrix

Table 1 presents the correlation matrix of all selected variables, which includes dependent variable (IOP), independent variables (DKC, IC, SOD), and controlling factor (ERI). The highest positive correlation (0.71) between IOP with IC was observed, followed by a strong positive correlation (0.62) between IOP and ERI. As for independent variables, the highest positive correlation (0.66) between DKC with ERI was observed, followed by a strong positive correlation (0.55) between ERI and SOD. The low correlations (0.26) between IC and DKC were also observed.

**Table 1:** Correlation matrix of dependent and independent variables.

	ERI	IC	IOP	DKC	SOD
ERI	1.00	0.66	0.26	0.40	0.31
IC	0.66	1.00	0.42	0.62	0.55
IOP	0.26	0.42	1.00	0.71	0.37
DKC	0.40	0.62	0.71	1.00	0.54
SOD	0.31	0.55	0.37	0.54	1.00

ERI, employees’ resistance to innovation; IC, innovation capability; IOP, innovative organizational performance; DKC, digital knowledge capability; SOD, strategic orientation towards digitalization.

### The qualities of measurements

**Figure 2:**
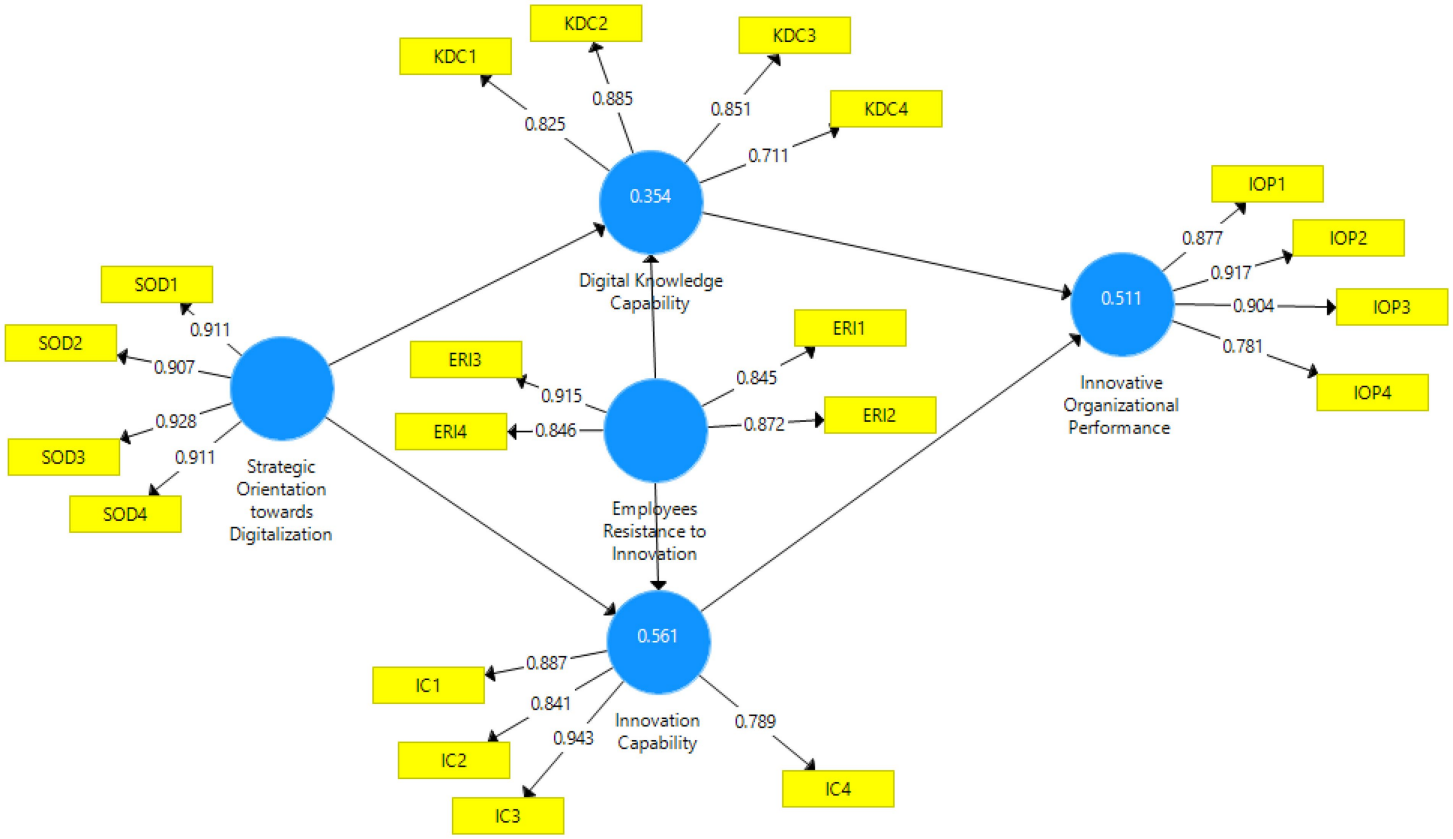
The *R*^2^ and factor loadings of each construct in the measurement model.

In terms of measuring the validity of each associated construct, we calculated its loading factor (LF), the average extracted variance (AVE), composite reliability (CR), and Cronbach alpha (α). Table 2 shows that all parameters are within the permissible range. Figure 2 illustrates the R2, path coefficients, and factor loadings of each construct in the developed model. The R2 of 0.51 was observed in model 1, meaning that the selected variables explain 51% of the variations.

**Table 2:** The qualities of measurements of selected items.

Items	LF	CR	AVE	α	VIF
DKC		0.89	0.67	0.84	
DKC1	0.83				1.86
DKC2	0.89				2.75
DKC3	0.85				2.35
DKC4	0.71				1.39
ERI		0.93	0.76	0.89	
ERI1	0.85				2.39
ERI2	0.87				2.27
ERI3	0.92				3.51
ERI4	0.85				2.28
IC		0.92	0.75	0.89	
IC1	0.89				3.25
IC2	0.84				2.93
IC3	0.94				4.71
IC4	0.79				1.78
IOP		0.93	0.76	0.89	
IOP1	0.88				2.52
IOP2	0.92				3.50
IOP3	0.90				3.34
IOP4	0.78				1.74
SOD		0.95	0.84	0.93	
SOD1	0.91				3.43
SOD2	0.91				3.27
SOD3	0.93				4.06
SOD4	0.91				3.46

ERI, employees’ resistance to innovation; IC, innovation capability; IOP, innovative organizational performance; DKC, digital knowledge capability; SOD, strategic orientation towards digitalization; LF, loading factor; AVE, the average extracted variance; CR, composite reliability; α, Cronbach alpha; VIF, the variance inflation factor.

Chaudhuri et al. (2022) stated that the PLS-SEM technique could not be carried out if the items do not explicitly describe their construct and do not explain that of the other things. As a result, it is necessary to confirm the discriminant validity of the test. Fornell and Larcker’s criterion Fornell and Larcker (1981) state that the square roots of all AVEs are more significant than the corresponding bifactor correlation coefficients, thus demonstrating discriminant validity. The Heterotrait-Monotrait (HTMT) test has been appropriately carried out to augment the Fornell and Larcker criteria for determining the validity of the discriminant analysis. Table 3 displays the findings of Fornell and Larcker’s criterion test. According to the findings, which are presented in Table 4, none of the HTMT values are higher than 0.85 (Voorhees et al., 2016). All variables have significant outcomes defined by Fornell and Larcker’s criterion.

**Table 3:** Discriminant validity test (Fornell and Larcker criterion).

	ERI	IC	IOP	DKC	SOD
ERI	0.87				
IC	0.66	0.87			
IOP	0.26	0.42	0.87		
DKC	0.40	0.62	0.71	0.82	
SOD	0.31	0.55	0.37	0.54	0.91

ERI, employees’ resistance to innovation; IC, innovation capability; IOP, innovative organizational performance; DKC, digital knowledge capability; SOD, strategic orientation towards digitalization.

**Table 4:** Heterotrait-Monotrait test (HTMT).

	ERI	IC	IOP	DKC	SOD
ERI					
IC	0.73				
IOP	0.28	0.47			
DKC	0.47	0.72	0.82		
SOD	0.34	0.60	0.40	0.61	

ERI, employees’ resistance to innovation; IC, innovation capability; IOP, innovative organizational performance; DKC: digital knowledge capability; SOD, strategic orientation towards digitalization.

### Structural equation model analysis

**Figure 3:**
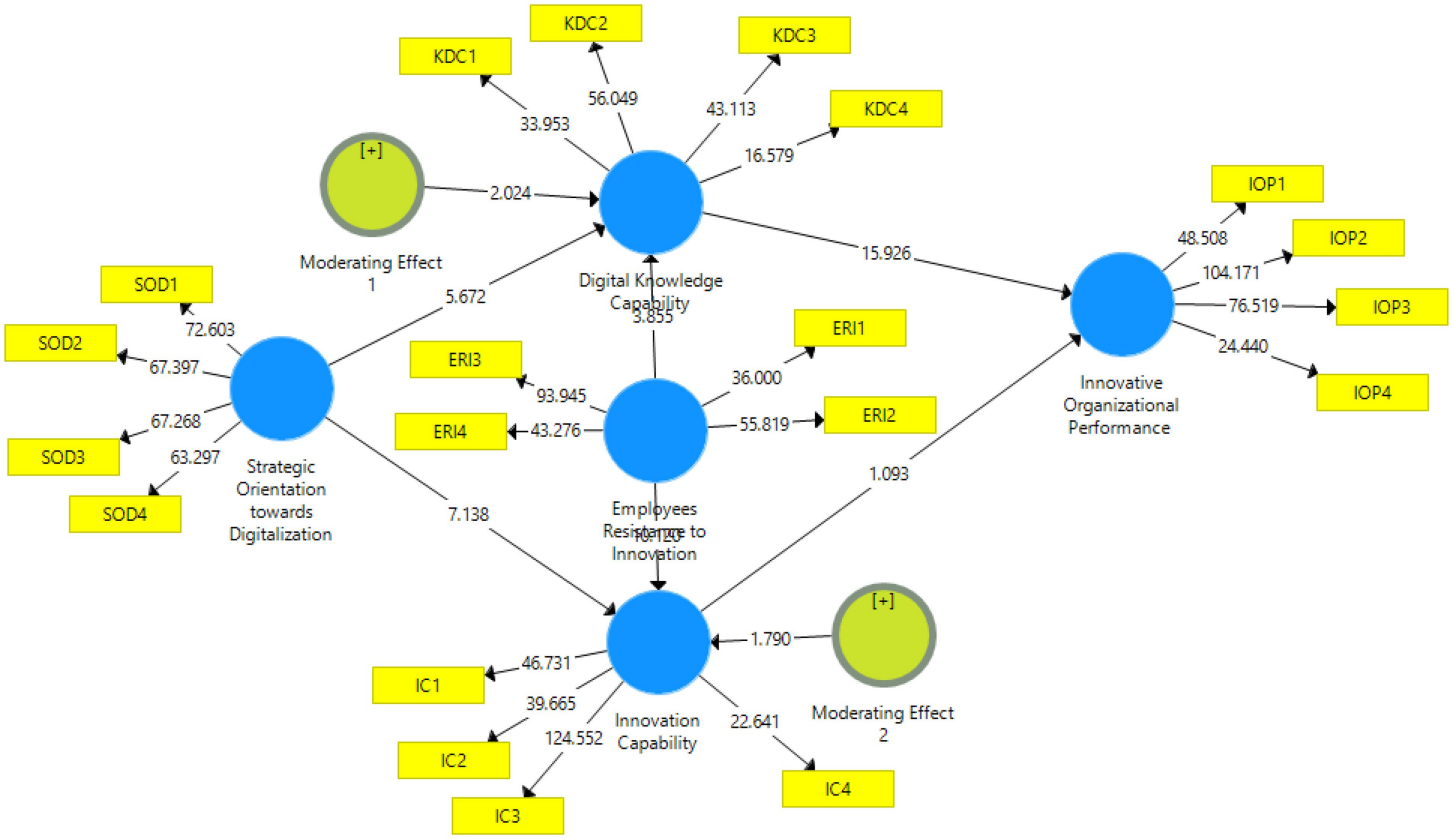
Structural model bootstrapping.

Using the PLS-SEM technique, the results confirm that each of the six hypotheses was correct. Two of the study’s six hypotheses pertain to moderator ERI’s effects on H5 and H6, respectively. A significant impact of SOD on DKC and IC (H1 and H2) is shown by the significant path coefficients, which both have t-values of 5.71 and 7.19, respectively. The significant impact of DKC (H3) on IOP is shown by the significant path coefficient, which has a t-values of 15.73. The path coefficient shows the significant impact of IC (H4) on IOP, which has a t-values of 2.10. For both H5 and H6, the ERI moderating effects on DKC and IC are considerable, with path coefficients t values of 3.85 and 10.35 determined to be statistically significant. The total effects of construct items are presented in Table 5, which significantly presents the sample mean, standard deviation, and *t* values, whereas Table 6 presents the f^2^ statistics of construct items. The *t*-values of each construct in developed model 2 are illustrated in Figure 3.

**Table 5:** Total effects of construct items.

	Sample mean	Standard deviation	*t* statistics	*p* values
ERI -> IC	0.53	0.05	10.35	0.00
ERI -> IOP	0.16	0.05	3.18	0.00
ERI -> DKC	0.24	0.06	3.85	0.00
IC -> IOP	−0.04	0.04	2.10	0.03
DKC -> IOP	0.74	0.05	15.73	0.00
Moderating Effect 1 -> IOP	−0.10	0.04	2.04	0.04
Moderating Effect 1 -> DKC	−0.13	0.06	2.06	0.04
Moderating Effect 2 -> IC	−0.07	0.04	2.21	0.07
Moderating Effect 2 -> IOP	0.13	0.04	2.01	0.04
SOD -> IC	0.35	0.05	7.19	0.00
SOD -> IOP	0.29	0.06	4.99	0.00
SOD -> DKC	0.41	0.07	5.71	0.00

ERI, employees’ resistance to innovation; IC, innovation capability; IOP, innovative organizational performance; DKC, digital knowledge capability; SOD, strategic orientation towards digitalization.

**Table 6:** F2 statistics of construct items.

	ERI	IC	IOP	DKC	SOD
ERI		0.60 (L)		0.26 (M)	
IC			0.28 (M)		
IOP					
DKC			0.68 (L)		
SOD		0.29 (M)		0.30 (M)	

ERI, employees’ resistance to innovation; IC, innovation capability; IOP, innovative organizational performance; DKC, digital knowledge capability; SOD, strategic orientation towards digitalization; L, large; M, medium.

### Analysis of The moderator

In this investigation, the ERI has been analyzed as a potential moderator, affecting the correlations investigated by H5 and H6. We utilized moderator analysis to investigate the impacts of ERI on H5 and H6. The study was carried out independently on each moderator category to determine how its presence affected the relationship (Nirino et al., 2022). Through the use of the bootstrapping method and the consideration of 5,000 resamples, this investigation confirmed the effects. Suppose the differences in the *p*-values of the moderator’s categories are either larger than 0.95 or less than 0.05. In that case, the effect of the moderator is regarded to have a substantial impact on the linkage (Hair et al., 2021). The findings indicate a statistically significant relationship between the moderator ERI and H5 and H6 (Table 5).

### Effect size statistics (f^2^)

The effect size f^2^ values need to be determined to determine whether or not the exogenous factors effectively contribute to the endogenous factors. F^2^ values that fall between 0.020 and 0.150 are considered to be weak (W); those that fall between 0.150 and 0.350 are considered to be medium (M), and those that fall beyond 0.350 are considered to be large (L; Wassertheil and Cohen, 1970). Table 6 presents the findings in their entirety.

### Hypotheses testing

To obtain an accurate estimate of the cross-validated redundancy measure concerning the dependent, the blindfolding process with an omission distance of 7 was utilized. According to the findings, the Stone-Geisser Q2 value was calculated to be 0.38, which indicates a favorable outcome (Chaudhuri et al., 2022). It demonstrates that the model has predictive relevance in a significant way.

This study’s findings suggest that employee resistance to innovation can hinder an organization’s effective implementation of a digital transformation strategy. In particular, this resistance may prevent us from fully realizing the potential benefits of such a strategy. The implications of these findings are significant, as they suggest that organizations seeking to implement a digital transformation strategy must first overcome any resistance to change among their employees (Albukhitan, 2020). Organizations must be aware of this resistance and take proactive steps to mitigate it to achieve their digital transformation goals. For example, training programs to educate and inform staff about innovation’s benefits should help lessen resistance to change. Managers should also actively promote innovation within their organization by engaging in dialogue about new ideas or introducing new processes or technologies into the workplace. Finally, organizations should consider implementing systems that reward those who innovate more than those who follow established rules without question. These initiatives will not only help stimulate creativity but also serve as a means of combating opposition to innovations within the company (Cichosz et al., 2020). As companies work to foster innovation, they must also work to address resistance to innovation and find ways to motivate all employees to embrace new approaches. Doing so will allow organizations to leverage digital transformation strategies’ benefits fully. Failing to do so will diminish their chances of success (Albukhitan, 2020; Cichosz et al., 2020; Trushkina et al., 2020).

The findings of this study are also important in the context of the industry because they highlight how resistance to innovation can hinder the positive effects of digitalization on organizational performance. In a rapidly changing and increasingly competitive business landscape, organizations need to be able to embrace new technologies and utilize them to their fullest potential (Soluk and Kammerlander, 2021). However, this can be difficult if there is resistance within the organization to change. This study’s findings suggest that for digitalization to improve organizational performance truly, organizations must first overcome any resistance to innovation. Only then can they develop a strategic orientation toward digitalization that will allow them to fully capitalize on its potential benefits (Burchardt and Maisch, 2019; Volberda et al., 2021). These findings have implications for both practitioners and academics alike. Practitioners should remember that only when an organization has established a healthy culture of innovation will they be able to take full advantage of digitalization’s opportunities. Academics should note the need for more research on what factors contribute to resistance to innovation to find effective ways to combat it before we lose out on all the possible benefits of embracing change (Vogelsang et al., 2019).

While some sources show that implementing a digital transformation strategy is beneficial and key to remaining competitive in today’s business environment, others warn about creating unrealistic expectations about its impact (Schneider and Kokshagina, 2021). For example, Parida et al. (2019) found that companies struggling with online customer service were likely to experience lower levels of innovativeness than those that embraced customer feedback through online channels. This again indicates how complex it can be to implement successful digital transformations in practice. A big part of this complexity is due to what Perry and Garud refer to as innovation inertia, which refers to resistance in the form of opposition or indifference to changes that challenge our worldview (Rodríguez-Abitia and Bribiesca-Correa, 2021; Schneider and Kokshagina, 2021; Soluk and Kammerlander, 2021). They argue that inertia stems from cognitive biases such as confirmation bias or overconfidence. One way to address these biases would be by increasing awareness among employees about these biases or by hiring employees who are less susceptible to these biases. Another would be creating a culture where being open-minded and thinking about other points of view is more important than being right or sure.

## Discussion

First, we computed the correlation matrix of all selected variables to see the association level among them. After that, we computed the quality of the measurements. Cronbach’s alpha coefficient was used to determine the reliability of all items, and an exploratory factor analysis was utilized to determine each item’s convergent and discriminant validity. There was no need to eliminate any item because the Cronbach alpha for all structures were more significant than 0.7 and acceptable (Adesta et al., 2018; Ahadiat and Dacko-Pikiewicz, 2020). Confirmatory factor analysis (CFA) was used to calculate the composite reliability and average variance extracted (AVE) for all the constructs in the study. When we performed a confirmatory factor analysis, we discovered that the majority of constructs had high reliability and uni-dimensionality, as evidenced by the fact that the composite reliability of each construct was more significant than 0.7 and the average variance explained by the construct was more significant than 0.5 (Ahadiat and Dacko-Pikiewicz, 2020).

In this work, strategic orientation theory has traditionally been used to identify successful ways to increase organizational performance. This paper’s goal was to use a well-known theoretical framework in a new setting so that the theory of digitalization may benefit from this research. Having examined the current state of digitalization activities in Norwegian companies, Sannes and Andersen (2017) have advocated for additional academic research into the subject. As a result of our research, we have gained new knowledge that will be discussed in light of our literature review. In the past, the theory of strategic orientation was mainly used to uncover practical ways of boosting corporate performance. Sannes and Andersen (2017) have studied companies’ digitalization efforts and urged further academic research on this topic. Because of this, we got a clearer picture of the issues at hand and formulated more in-depth hypotheses to test our theories. The findings reveal that digital distinctiveness is also influenced by strategic direction, one of the two digitalization aspects. Both market and technology orientations have a favorable impact on profitability.

The findings support the study by Sannes and Andersen (2017), which says that organizations must change to meet customers’ wishes to use digitalization for commercial purposes. Some academics assert that technology-focused companies devote more resources to innovation than those that are not. According to experts, there is no difference between the two organizations in the digital age that combine technology and business (SANNES and Andersen, 2017). According to several studies, entrepreneurialism has a favorable impact on digital distinction. The department’s emphasis on digital technology leads to a focus on efficiency. As institutional theory suggests, companies that compete in the same market tend to become more similar over time. Managers are constantly observing and imitating developments from other organizations that they believe will benefit their own. Isomorphism describes the development of similar-looking organizations due to this adaptability. Isomorphism refers to organizations in a particular field’s tendency to adopt similar structures to compete. Automating and simplifying processes may be necessary to succeed in the market (Fletcher et al., 2020; Isensee et al., 2020; Satalkina and Steiner, 2020).

When it comes to increasing consumer adoption of new digital technologies, the aspects in the second model are more important to present a company’s worldwide image or deal with negative word of mouth about digital advances. Diffusion of new ideas, marketing, and strategy are all incorporated into the findings. Identifying the elements that influence and increase the likelihood of adoption of digital innovation is essential to the diffusion of the innovation field, and this study’s findings do just that. Firms can speed up their technology adoption if they investigate and understand the factors that lead to late adoption. Because of awareness of the adopter profile, technology companies may build products that appeal to both early adopters and those who are more established (Burchardt and Maisch, 2019; Parida et al., 2019; Vogelsang et al., 2019; Volberda et al., 2021). Firms can cover the whole diffusion curve of innovation if they develop these technologies. Firms that do not adopt new technologies do not come up with new ideas compared to adopters or lead users. In other words, they may be members of current user communities, where negative word of mouth is the norm when spreading the news about new digital breakthroughs (Albukhitan, 2020; Trushkina et al., 2020; Soluk and Kammerlander, 2021). Customers will develop a positive attitude toward the technology if they have a strong relationship with the company. It will also allow corporations to lessen the negative word of mouth about their technology, which will, in turn, help to speed up the adoption of that technology. Hence, advertisements, trials, and positive word of mouth should be used to demonstrate the worth of a company’s technology (Parida et al., 2019; Rodríguez-Abitia and Bribiesca-Correa, 2021; Schneider and Kokshagina, 2021).

Organizations must have a clear and concise digitalization strategy as the business world becomes increasingly digitized. However, a new study has found that many organizations resist innovation, hindering their ability to develop a successful digitalization strategy (Borowski, 2021). The most common reasons managers resist innovation are fear of being unable to keep up with technological changes, fear of failure, and fear of change. Interestingly, the researchers discovered this is true for large companies; smaller businesses face these same challenges when trying to adapt (Casalino et al., 2020). When implementing a digitalization strategy for your organization or company, it’s important to know how these factors might hinder progress or otherwise hamper your efforts. Fortunately, there are ways to overcome some obstacles (Parida et al., 2019). For example, if someone is worried about keeping up with changing technology or risk-taking without knowing whether they will succeed or fail, think about developing a trial period for your innovations and using benchmarking against other competitors to gauge success rates.

Additionally, while overcoming fear of change and rejection is already discussed, remember that not everyone will embrace the innovations. Embrace both success and failure because we can learn something from them both. Remember: a good digitalization strategy starts at the top. Make sure senior management understands what needs to happen, who should take charge of different tasks (such as budgeting), who will oversee those tasks, and so forth. The chances for success will greatly increase with everyone on board with the plan and armed with the right tools.

This is not just an issue faced by multinational corporations, either. Even small businesses struggle to stay competitive in today’s fast-paced market (Sawy et al., 2016; Borowski, 2021). Larger companies may have big marketing departments, big R&D labs, or a well-established hierarchical structure in a chart. Still, smaller companies can be even more disadvantaged because they do not have as easy access to resources like capital and labor.

## Conclusion

Innovation is essential for businesses to maintain a competitive edge and keep up with the ever-changing landscape. However, many organizations face resistance to innovation, which can hinder their ability to adopt new technologies and practices. This resistance can come from various sources, such as a lack of understanding of the benefits of innovation or a fear of change. Overcoming these barriers is essential for organizations that want to improve their performance and stay ahead of the curve. One way to do this is by aligning their strategic orientation with digitalization efforts. This allows businesses to understand how new technologies can be used to achieve their goals and create value for their customers. A shift in strategy will help them move forward while adapting to changes in the industry.

### Strategic implications

We found that having either a commercial or technological orientation is beneficial to digital distinctiveness. According to this finding by researchers, companies should analyze the components of these two approaches and consider adapting some of their qualities to maximize their commercial exploitation of digitalization. According to our findings, market orientation had the greatest positive impact on digital distinction. Some organizations may already have a competitive advantage or be successful without implementing digital technologies. On the other hand, some organizations may already have the resources to implement digital solutions independently.

Digitalization has become the key to achieving a competitive advantage in many organizations’ highly competitive business landscape. However, digitalization also brings various risks that require IT departments to be agile and flexible to handle them effectively and efficiently. According to research, 90% of enterprises implement digital strategies to achieve a competitive advantage in this market (Leeflang et al., 2014). Yet, how these strategies are implemented determines their success or failure. A strategic orientation towards digitalization can improve the business’s chances of success through more effective and efficient handling of technological changes and opportunities. In a rapidly digitalizing world, organizations need to be strategic about their orientation to stay ahead of the curve and remain competitive. A strategic orientation helps organizations identify opportunities and threats in the marketplace and decide how to utilize the best digital tools and resources (Kessler and Chakrabarti, 1996; Kickul and Gundry, 2002; Kapidani et al., 2020).

Additionally, a strategic orientation can help organizations build a shared understanding of digitalization within the company and align employees around a common goal. Ultimately, a strategic orientation toward digitalization is essential for any organization that wants to stay relevant in today’s economy (Laforet, 2008; Kusiak, 2009). It enables businesses to identify opportunities and threats in the marketplace and take advantage of all available digital tools. A strategic orientation also encourages clear communication among employees so they are on the same page when utilizing new technologies or addressing issues related to change management. These four benefits of having a strategic orientation toward digitalization have helped many companies use technology to its fullest potential (Narver and Slater, 2012; Leeflang et al., 2014; Niemand et al., 2017; Munim and Noor, 2020).

Organizations must consider utilizing digital resources to achieve their desired outcomes in a rapidly digital world. The proper strategic orientation can help an organization gain a competitive edge in the marketplace. It is essential for organizations first define what they want to accomplish with digitalization before choosing which strategy will work best for them. Market-oriented organizations focus on developing new products or services through continuous feedback from the customers (Robbins and Barnwell, 2006; Niemand et al., 2021). Resource-oriented organizations have different goals; they invest time and money into researching new technologies or expanding current product lines to maintain a competitive advantage over other companies (SANNES and Andersen, 2017). Finally, learning-oriented organizations focus on improving their processes by integrating digital technology into everyday operations. A practical strategic orientation does not just happen by itself; it depends on each department in the company using it as a guide when making decisions about how to use new technologies, data collection methods, etc.

Furthermore, employees must know what each type of orientation entails to make educated decisions when adopting them (Satalkina and Steiner, 2020; Talwar et al., 2020). For example, suppose a resource-oriented organization invests too much in research and development without ever delivering anything to the market. In that case, they may find themselves going out of business quickly. Learning-oriented organizations must carefully examine which digital tools will improve their production capabilities and not merely replace labor (Voorhees et al., 2016). As these examples show, there is no one size fits all approach to digitalization; instead, many options are available depending on organizational needs. Once managers decide which type of orientation is most appropriate for their organization, they should select the corresponding subtype based on how deeply involved in digitalization they want to be (Zhou et al., 2005). If a firm wants to develop new products or services based on consumer insights, it would be most suitable to adopt a market-oriented strategy. However, some firms may prefer more passive techniques such as monitoring social media trends instead of diving headfirst into digitalization (Zhou et al., 2005; Voorhees et al., 2016).

### Limitations and future directions

The findings of this study are valuable, but they also have several limitations. The sample comprised people who were logged in *via* a browser. Future studies should investigate the impact of all six characteristics indicated in this study on adopting other digital breakthroughs, such as more recent breakthrough innovations, to confirm the generalizability of the conclusions. Data from one country was selected as a random sample for the study. Future research should investigate if age, culture, and geography have a role in the diffusion of digital advances by conducting a cross-national study. Digital innovation is a primary focus for governments nowadays. To encourage and speed up the use of digital advances in future research, governments, and public policymakers may need to help. Finally, for startups, established enterprises, and government organizations alike, understanding the phenomenon of late adoption and the late adopter profile can be critical to the production, development, and implementation of digital innovations. The results help the innovation and new product development field by looking at adoption and what drives it and giving ideas on how to increase the rate at which digital innovations are adopted.

## Data availability statement

The original contributions presented in the study are included in the article/Supplementary material, further inquiries can be directed to the corresponding author.

## Ethics statement

The studies involving human participants were reviewed and approved by Xian University of Finance and Economics, China. The patients/participants provided their written informed consent to participate in this study. The study was conducted in accordance with the Declaration of Helsinki.

## Author contributions

The author confirms being the sole contributor of this work and has approved it for publication.

## Funding

Research on innovation efficiency evaluation of Equipment Manufacturing industry in Shaanxi Province based on Exploratory Spatial Analysis (18JZ029), Supported by Philosophy and Social Science Key Research Base project of Shaanxi Education Department; Green Innovation Performance Evaluation and Spatial Difference Evolution in Shaanxi Province (2021R008), Supported by Shaanxi Social Science Foundation project.

## Conflict of interest

The author declares that the research was conducted in the absence of any commercial or financial relationships that could be construed as a potential conflict of interest.

## Publisher’s note

All claims expressed in this article are solely those of the authors and do not necessarily represent those of their affiliated organizations, or those of the publisher, the editors and the reviewers. Any product that may be evaluated in this article, or claim that may be made by its manufacturer, is not guaranteed or endorsed by the publisher.
